# A biochemical study on ameliorative effect of green tea (Camellia sinensis) extract against contrast media induced acute kidney injury

**DOI:** 10.12861/jrip.2014.16

**Published:** 2013-11-09

**Authors:** Hamid Nasri, Ali Ahmadi, Azar Baradaran, Parto Nasri, Shabnam Hajian, Armita Pour-Arian, Golnoosh Kohi, Mahmoud Rafieian-Kopaei

**Affiliations:** ^1^Department of Nephrology, Division of Nephropathology, Isfahan University of Medical Sciences, Isfahan, Iran; ^2^Department of Epidemiology, Shahid Beheshti University of Medical Sciences, Tehran, Iran; ^3^Department of Pathology, Isfahan University of Medical Sciences, Isfahan, Iran; ^4^Nickan Research Institute, Isfahan, Iran; ^5^Medical Plants Research Center, Shahrekord University of Medical Sciences, Sharekord, Iran

**Keywords:** Contrast media, Green tea, Renal injury, Nephrotoxicity

## Abstract

**Introduction:** Reactive oxygen species have been shown to be mediators of kidney injury and green tea polyphenols are potent-free radical scavengers.Objectives: In this study we sought to examine whether green tea was able to protect renal toxicity induced by contrast media or not.

**Materials and Methods:** In this experimental study 40 rats were randomly divided into four groups including: 1) control group 2) contrast media group 3) contrast media plus green tea 4) Green tea pretreatment and contrast media group. Blood urea Nitrogen (BUN) and serum creatinine were assessed for severity of kidney injury.

**Results:** Serum creatinine level was higher in group II than in other groups (p<0.001). Treatment (group 3) or pretreatment (group 4) with green tea significantly reduced blood creatinine level when compared with contrast media group (group 2).

**Conclusion:** In this study, beneficial property of green tea, against renal toxicity of contrast media was observed. Green tea extract is an inexpensive, nontoxic, and effective treatment modality in individuals with a risk for acute kidney injury of contrast media.

**Implication for health policy/practice/research/medical education:**

In an experimental study on 40 rats beneficial property of green tea (Camellia sinensis) extract, against renal toxicity of contrast media was observed. Green tea extract is an inexpensive, nontoxic, and effective treatment modality in individuals with a risk for acute kidney injury of contrast media.

## Introduction

Contrast media-induced acute renal damage accounts for approximately 10% of all causes of hospital-acquired kidney failures, and may cause prolong in-hospital stay, and represents a strong predictor of poor early and late outcome (1). Following intravascular administration, contrast media (CM) is circulated rapidly in intravascular and extracellular fluids (1,2). They are removed only by glomerular filtration. In individuals with normal kidney function, CMs are excreted within 24 h. The pathophysiology of contrast media-induced acute renal damage is based on three separate but interacting mechanisms: formation of reactive oxygen species (ROS), direct tubular cell toxicity and medullary ischemia. The nephrotoxicity of CM is revealed by increase in serum creatinine level (1-3). In fact, CM-induce renal injury is the third most common cause of renal dysfunction (1-3). Recently much attention has been made on the efficacy of medicinal plants in prevention and treatment of various diseases, especially in kidney and liver complications. Most of these beneficial effects of plants are attributed to their antioxidant properties (4,5). Green tea (GT), prepared from the leaves of Camellia sinensis L., is a popular beverage worldwide. Polyphenols in GT have been receiving much attention as potential compounds for the treatment of various diseases with low toxicity (4-6). Especially, its antioxidant properties in protection of renal injury caused by oxidative stress have been on focus.

## Objectives

In this study, we sought to investigate the ameliorative effects of GT against the renal injury induced by contrast media.

## Materials and Methods

### Animals

The present study included 40 adult male Wistar rats (six weeks old) with a mean body weight of 200-250g. In this experimental design, rats were designated randomly into four experimental equal groups as follow:

Group I: control group (sham group); they were not received any drugs.
Group II: contrast media group; they received iodixanol 10 ml/kg/single dose by i.v. injection.
Group III: contrast media and green tea; rats in this group, firstly received iodixanol 10 ml/kg/single dose by i.v. injection, then they treated with green tea 10 ml/kg/day by i.p. injection on days 3, 4 and 5.
Group IV: green tea and contrast media group; rats in this group were pretreated with green tea 10 ml/kg/day by i.p. injection on days 1, 2 and 3, then they received iodixanol 10 ml/kg by i.v. injection.

### Experimental study protocol

The experiment protocol was approved by Ethical Committee of Shahrekord University of Medical Sciences (Ethical Code No: 91/11/1). All rats were given unlimited access to standard rat chow and water. On the first day, blood samples (1 ml) were collected from the lateral tail vein for examination of creatinine (Cr) and blood urea nitrogen (BUN) levels. After 20 min, rats of group IV were given 10 ml/kg of green tea by daily i.p. injection for 3 days. On the third day, 10 ml/kg contrast media was injected to animals in groups II and IV via the lateral tail vein. After 20 min rats of group III were given 10 mg/kg of green tea by i.p. injection daily for 3 days. On the fifth day, all rats were anesthetized and the blood samples were collected for evaluation of Cr and BUN levels and then all rats were killed using ketamin.

### Data analysis

All parameters were summarized with mean and standard deviation. One-way Analysis of Variance (ANOVA) and post hoc Bonferroni test were used for the comparison of mean values between groups. P< 0.05 were assumed to be significant. Data was analyzed by Stata (Stata corp, College Station, Tex).

## Results

Mean and standard deviation of creatinine and BUN in each group and total groups are shown in Table 1 .

**Table 1:** Mean ±SD of creatinine and BUN in each group and total groups.

**Groups**	**Creatinine**		**BUN**	
	**Mean**	**SD**	**Mean**	**SD**
Control (Group I )	0.59	0.5	25.3	1.3
Contrast media (Group II)	1.65	1	27.4	0.5
Contrast media+ Green tea (Group III)	0.68	0.7	23.7	8
Green tea, then Contrast media (Group IV)	0.61	0.6	22.1	3
Total	0.88	0.1	24.6	0.7

Mean of creatinine between groups was significant and shown by F Statistic (ANOVA) in Table 2 .

**Table 2:** Comparison of mean value of biochemical factors between groups by F Statistics (ANOVA) and P Value.

**Comparison Mean Value Between Groups**	**F Statistics** ** df=39**	**P**
BUN	2.51	0.07
Creatinine	66.8	0.001*

*P< 0.05 were assumed to be significant.

Creatinine levels were significantly higher in the group II than in the control group, III and IV groups (p=0.001). Difference creatinine values and comparison them, between groups are shown in Table 3 .

**Table 3:** Difference creatinine values and comparison of creatinine between groups.

**Comparison between groups**	**Difference creatinine Values**
**I** vs. **II**	1.06 0.001*
**I** vs. **III**	0.09 0.99
**I** v.s **IV**	0.02 0.9
**II** v.s **III**	0.97 0.001*
**II** v.s **IV**	1.04 0.001*
**III** v.s **IV**	0.07 0.9

*P< 0.01 were assumed to be significant.

Treatment or pretreatment with green tea significantly reduced blood creatinine level when compared with control group.

## Discussion

In this study the beneficial effect of green tea against nephrotoxicity of contrast media was shown. Creatinine level in intervention group with contrast media (group II) was more than other groups and Green tea reduced the level of creatinine in this experimental study. Abdel-Raheem *et al*, showed the protective effect of GT on renal oxidative damage induced by gentamicin in rats. They found, the administration of GT plus gentamicin protected renal tissues against renal toxic effect of gentamicin as observed by improvement of histopathological alterations and normalization of renal biochemical parameters (7). Similarly, Rehman *et al*. showed that GT polyphenols markedly diminished cyclosporine-induced renal damage and improved renal function (8). Amelioration of renal injury by GT was also showed by Ryu *et al*. against cyclosporine nephropathy (9) and also in the study conducted by Shin *et al* (10). They observed that GT had considerable antiproteinuric property through antioxidative activity from nephropathy of cyclosporine (9,10). It is well understood that green tea polyphenols have a beneficial property on pathological states related to oxidative stress of renal tissues (4-7). Also, green tea polyphenols have been shown to prevent the metal-catalyzed formation of radical species, antioxidant enzyme modulators, and scavengers of free radicals, including the hydroxyl radical (4-7). In our investigation, beneficial property of GT against contrast induced acute kidney injury was observed. However, more studies are necessary to find out whether pretreatment of GT against contrast induced kidney injury is more effective than co-administration of this herb with contrast media. In fact, GT can provide an economical, nontoxic, and applicable intervention approach in individuals with a risk for contrast induced acute kidney injury. In this regard, further preclinical and human investigations are necessary to complete the data from other studies in efficiency of this herb against various injurious substances to the renal tubular cells.

## Authors’ contributions

All authors contributed in design of the research. AH analyzed the data. HN, GK, SH, AP, PN and AB wrote the manuscript. MRK edited the paper. All authors read and approved the paper.

## Conflict of interests

The authors declared no competing interests.

## Ethical considerations

Ethical issues (including plagiarism, data fabrication, and duplicate publication) have been completely observed by the authors.

## Funding/Support

This paper has been derived from the residential thesis. This study was granted by Shahrekord University of Medical Sciences (grant number: 1395). The study was conducted in Medical Plants Research Center of Shahrekord University of Medical Sciences.

## References

[1] McDonald RJ, McDonald JS, Bida JP, Carter RE, Fleming CJ, Misra S (2013). Intravenous contrast material-induced nephropathy: causal or coincident phenomenon. Radiology.

[2] Maliborski A, Żukowski P, Nowicki G, Bogusławska R (2011). Contrast-induced nephropathy—a review of current literature and guidelines. Med Sci Monit.

[3] Yat wong P, LI Z, Guo J, Zhang A (2012). Pathophysiology of contrast-induced nephropathy. Int J Cardiol.

[4] Rafieian-Kopaie M, Baradaran A (2013). Plants antioxidants: From laboratory to clinic. J Nephropathol.

[5] Rafieian-Kopaei M (2012). Medicinal plants and the human needs. J HerbMed Pharmacol.

[6] Asadi SY, Parsaei P, Karimi M, Ezzati S, Zamiri A, Mohammadizadeh F (2013). Effect of green tea (Camellia sinensis) extract on healing process of surgical wounds in rat. Int J Surg.

[7] Abdel-Raheem IT, EL-Sherbeny GA, Taye A (2010). Green tea ameliorates of renal oxidative damage induced by gentamicin in rats. Pak J Pharm Sci.

[8] Rehman H, Krishnasamy Y, Haque K, Thurman RG, Lemasters JJ, Schnellmann RG (2013). Green tea polyphenols stimulate mitochondrial biogenesis and improve renal function after chronic cyclosporin a treatment in rats. PLoS One.

[9] Ryu HH, Kim HL, Chung JH, Lee BR, Kim TH, Shin BC (2011). Renoprotective effects of green tea extract on renin-angiotensin-aldosterone system in chronic cyclosporine-treated rats. Nephrol Dial Transplant.

[10] Shin BC, Kwon YE, Chung JH, Kim HL (2012). The antiproteinuric effects of green tea extract on acute cyclosporine-induced nephrotoxicity in rats. Transplant Proc.

